# Early and Pre-Clinical Detection of Prion Seeding Activity in Cerebrospinal Fluid of Goats using Real-Time Quaking-Induced Conversion Assay

**DOI:** 10.1038/s41598-019-42449-7

**Published:** 2019-04-16

**Authors:** Alessandra Favole, Maria Mazza, Elena Vallino Costassa, Antonio D’Angelo, Guerino Lombardi, Paola Marconi, Paola Crociara, Elena Berrone, Marina Gallo, Claudia Palmitessa, Christina D. Orrù, Byron Caughey, Pier L. Acutis, Maria Caramelli, Cristina Casalone, Cristiano Corona

**Affiliations:** 1 National Reference Laboratory of TSEs (CEA), Istituto Zooprofilattico Sperimentale del Piemonte, Liguria e Valle d’Aosta, Turin, Italy; 2 Dipartimento di Scienze Veterinarie, Sezione Clinica Medica, University of Turin, Grugliasco, Turin, Italy; 3 Istituto Zooprofilattico Sperimentale della Lombardia e dell’Emilia Romagna, Brescia, Italy; 4 Istituto Zooprofilattico Sperimentale Lazio e Toscana, Firenze, Italy; 5 Rocky Mountain Laboratories, National Institute for Allergy and Infectious Diseases, National Institutes of Health, Hamilton, Montana, USA

## Abstract

Since 2005, two cases of natural bovine spongiform encephalopathies (BSE) have been reported in goats. Furthermore, experimental transmissions of classical (C-BSE) and atypical (L-BSE) forms of BSE in goats were also reported. To minimize further spreading of prion diseases in small ruminants the development of a highly sensitive and specific test for *ante-mortem* detection of infected animals would be of great value. Recent studies reported high diagnostic value of a second generation of cerebrospinal fluid (CSF) Real-Time Quaking-Induced Conversion (RT-QuIC) assay across a wide spectrum of human prions. Here, we applied this improved QuIC (IQ-CSF) for highly efficient detection of TSEs prion protein in goat cerebrospinal fluid. IQ-CSF sensitivity and specificity were evaluated on CSF samples collected at disease endpoint from goats naturally and experimentally infected with scrapie or bovine isolates of C-BSE and L-BSE, respectively. Next, CSF samples collected from L-BSE infected goats during pre-symptomatic stage were also analysed. PrP^L-BSE^ associated seeding activity was detected at early time points after experimental inoculation, with an average time of 439 days before clinical symptoms appeared. Taken together these data are indicative of the great potential of this *in vitro* prion amplification assay as *ante-mortem* TSE test for live and asymptomatic small ruminants.

## Introduction

Transmissible Spongiform Encephalopathies (TSEs) or prion diseases are fatal neurodegenerative diseases that include Creutzfeldt-Jakob disease (CJD) in humans, bovine spongiform encephalopathy (BSE) in cattle, scrapie in sheep and chronic wasting disease (CWD) in cervids. The infectious agent responsible for these diseases, the prion, appears to be composed primarily of an abnormal, misfolded and oligomeric (PrP^Sc^) form of the cellular prion protein (PrP^C^)^1,2^. PrP^Sc^ induces the polymerization and conformational conversion of PrP^C^ to its infectious form^3–5^ or, in a variety of *in vitro* conversion assays^6^, into alternative forms of the prion protein which are, similarly to PrP^Sc^, partially resistant to digestion with proteases (PrP^Res^). The pathogenic isoform of the prion protein is therefore a marker associated to TSEs which acts as a *seed* allowing the ultrasensitive detection of PrP^Sc^ using *in vitro* prion amplification reactions such as Real-Time Quaking Induced Conversion (RT-QuIC)^7^.

The spread of BSE agent to small ruminants is a major issue in the surveillance of TSEs because BSE passage into a new host may change strain properties, make it difficult to recognize the original strain, and increasing the risk of epidemic spread^8^. Since 2005, two natural BSE cases have been reported in goats^9,10^. Furthermore, experimental transmissions of classical (C-BSE) and atypical (L-BSE) forms of BSE in goats were also reported^11^. An important goal to minimize further spreading of small ruminant TSEs is the development of an assay for highly sensitive and *intra-vitam* detection of prions in infected, but not clinically sick animals. Currently, small ruminant TSEs are definitively diagnosed by *post-mortem* examinations of brain stem to detect PrP^Sc^ ^12^. RT-QuIC assays can rapidly detect sub-infectious levels of prion seeding activity and have been used successfully to detect multiple human, cervid, ovine, hamster, and mouse prion strains in a variety of biological tissues, such as skin^13^, cerebrospinal fluid^14–18^, saliva^19^, blood^20–23^, and nasal brushings^24^, showing that this test has the potential of being used for *ante-mortem* TSE diagnosis. Important studies on human cerebrospinal fluid from patients with sporadic Creutzfeldt-Jakob disease (sCJD) have shown that RT-QuIC has the potential to improve *ante-mortem* diagnosis for this disease, with a high degree of specificity^15–17,25–29^. Results obtained by several laboratories with the first generation of this assay (referred to here as previous QuIC-CSF [PQ-CSF]), mainly using full-length (23–231) hamster recombinant PrP (rPrP^Sen^) as the substrate, demonstrated a very high specificity but a suboptimal sensitivity, especially in sCJD subtypes associated with PrP^Sc^ type 2^30,31^. However, Orrù *et al*.^25^ recently introduced an improved, second generation RT-QuIC for CSF (referred to here as improved QuIC-CSF [IQ-CSF]) assay which uses a truncated form of hamster rPrP^Sen^ (amino acids 90–231) as the substrate, higher incubation temperatures, and the addition of SDS. Initial evaluation of the IQ-CSF assay indicated greater analytical and diagnostic sensitivity, and markedly shorter testing times^25–29^.

Studies have demonstrated that CSF tests that measure alterations in total PrP levels and prion seeding activity by RT-QuIC, may be useful in the identification of pre-clinical prion cases^20,32^. Still, it remains important to understand the time course of prion seeding activity accumulation in cerebrospinal fluid of prion-infected small ruminants in order to develop an early, sensitive, and specific test for *ante-mortem* and pre-clinical detection of PrP^Sc^. Furthermore, to date there is no information on experimental conditions to amplify goat BSE prion strains by RT-QuIC. Moreover, IQ-CSF conditions have not been previously applied to animal CSF samples. Here, we report for the first time sensitive detection of different small ruminant prion strains in brain and CSF of TSE-infected goats by RT-QuIC assays. Furthermore, *intra-vitam* detection of preclinical L-BSE-infected goats at early time points is also described. Taken together these data are indicative of the great potential of this *in vitro* prion amplification assay as *ante-mortem* TSE test for live and asymptomatic small ruminants.

## Results

### RT-QuIC seeding activity in brains from natural scrapie and experimental C-BSE- and L-BSE-infected goats

**Figure 1:**
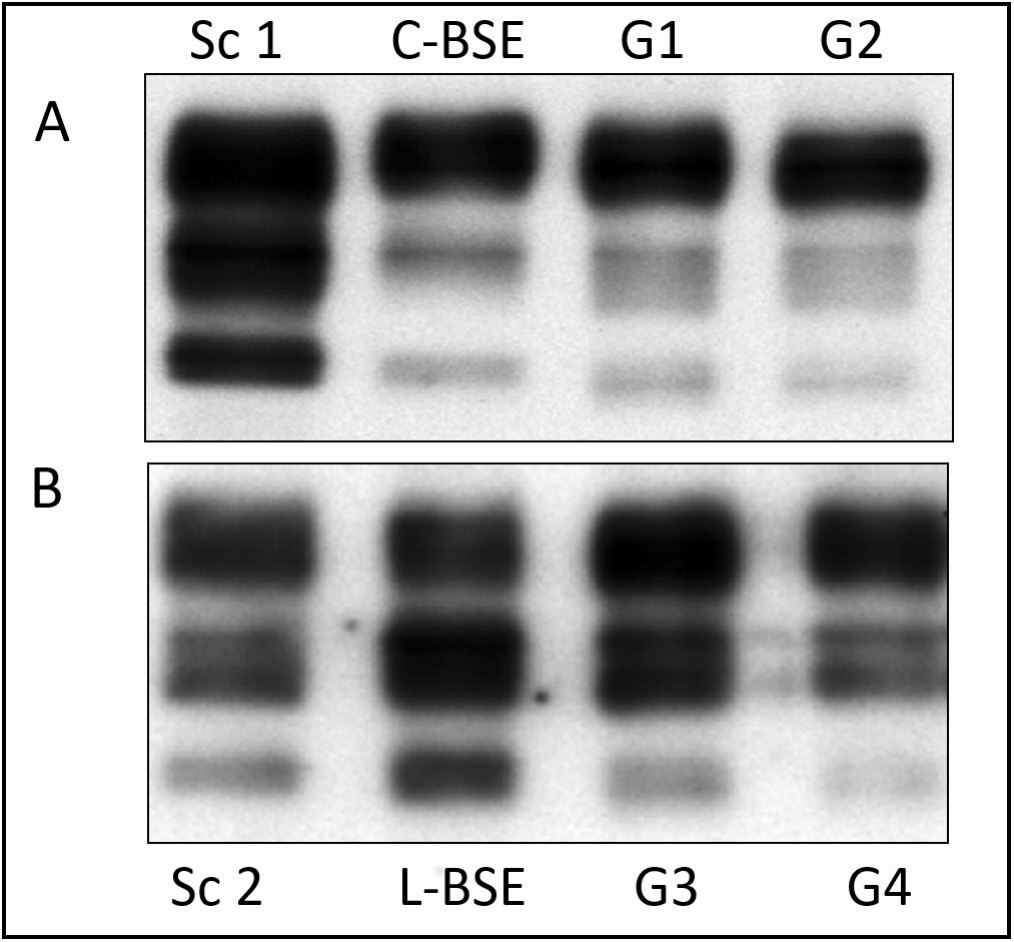
Western blot analysis of brainstem of goats inoculated with C- or L-Type BSE. (**A**) PrP^Sc^ was extracted from the brainstem of two representative goats inoculated with C-type BSE (G1, G2). Brain tissue from cattle C-BSE, used for the inoculum, and previously confirmed classical scrapie goat (Sc 1) served as positive controls. (**B**) PrP^Sc^ was extracted from the brainstem of two representative goats inoculated with L-type BSE (G3, G4). Brain tissue from cattle L-BSE, used for the inoculum, and previously confirmed classical scrapie goats (Sc 2) served as controls. All samples were treated with Proteinase K. Applied tissue equivalents were 3 mg (C-BSE goats) or 8 mg (L-Type BSE goats). Membranes were probed with mAb SAF 84. Full length blots including negative controls and all goats inoculated with C- or L-type BSE are presented in Supplementary Figs S1 and S2, respectively.

To test the presence of PrP^Sc^ before proceeding to subsequent RT-QuIC analyses, Western Blot (WB) analyses were carried out on brainstem collected from experimental C-BSE and L-BSE infected goats (Table 1), recently characterized in our work by Vallino Costassa *et al*.^11^. Brain tissue from cattle C-BSE and L-BSE samples, used for the inoculum, and previously confirmed classical scrapie goats served as controls. Western Blot analyses revealed strong immunosignals related to the presence of pathological prion protein in all analysed samples (Fig. 1).

**Table 1:** Study Populations.

ID	Goat PrP	Breed	Age (years)	Route of infection	Genotype	CNS PrP^Sc^	PrP^Sc^ peripheral distribution
G1	C-BSE	Saanen	2	IC	ARQ/ARQ; 240 P/P	+	subm.ln, retroph.ln, spleen
G2	C-BSE	Saanen	2	IC	ARQ/ARQ; 240 P/P	+	subm.ln, retroph.ln, par. ln, icv, spleen
G3	L-BSE	Saanen	3	IC	ARQ/ARQ; 240 P/P	+	−
G4	L-BSE	Saanen	3	IC	ARQ/ARQ; 240 P/P	+	−
G5	L-BSE	Saanen	3	IC	ARQ/ARQ; 240 P/P	+	−
G6	L-BSE	Saanen	3	IC	ARQ/ARQ; 240 P/P	+	−
G7	L-BSE	Saanen	3	IC	ARQ/ARQ; 240 S/P	+	−
G8	L-BSE	Saanen	3	IC	ARQ/ARQ; 240 P/P	+	−
G9	C-BSE	Saanen	2	IC	ARQ/ARQ; 240 S/P	+	subm.ln, retroph.ln, par. ln, icv, spleen
G10	C-BSE	Saanen	2	IC	ARQ/ARQ; 240 P/P	+	subm.ln, retroph.ln,tonsils
G11	C-BSE	Saanen	2	IC	ARQ/ARQ; 240 P/P	+	n. a.
Sc 1	scrapie	Mixed Breed	5	Natural	222 Q/Q	+	n. a.
Sc 2	scrapie	Mixed Breed	2	Natural	222 Q/Q	+	n. a.
CTR 1	None	Mixed Breed	5	−	ARQ/ARQ; 240 P/P	−	−
CTR 2	None	Saanen	2	−	ARQ/ARQ; 240 P/P	−	−

Breed, age, route of prion infection, genotype, CNS PrP^Sc^ and PrP^Sc^ peripheral distribution. IC = *intracranial*; n. a. = not available.

**Figure 2:**
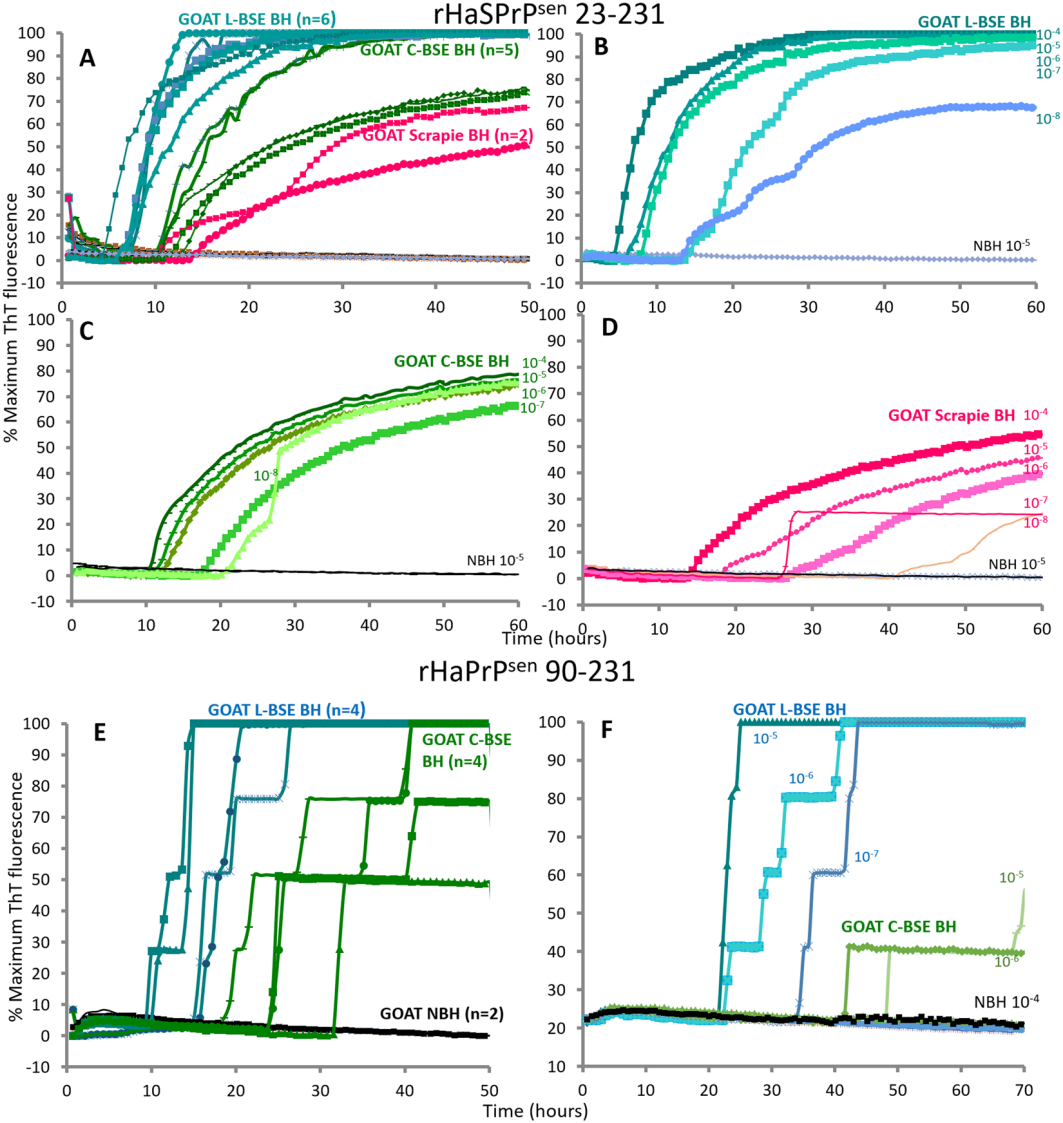
RT-QuIC sensitive detection of PrP^L-BSE^, PrP^C-BSE^ and PrP^s^^crapie^ from goat brain homogenates. (**A**) rHaSPrP^Sen^ 23–231 substrate was used to detect both PrP^L-BSE^ (azure), PrP^C-BSE^ (green) and PrP^scrapie^ (magenta) from brain homogenates. 10^−4^ brain tissue dilutions were used to seed quadruplicate RT-QuIC reactions. Normal control brain homogenates (black) showed no response. The number of samples is in parentheses. Representative sensitivity of detection for PrP^L-BSE^ (shades of azure; **B**) PrP^C-BSE^ (shades of green; **C**) and PrP^s^^crapie^ (shades of magenta; **D**) in brain homogenates (BH) using rHaSPrP^Sen^ 23–231 as a substrate. Dilutions are indicated next to the curve. (**E**) rHaPrP^Sen^ 90–231 substrate was used to detect both PrP^L-BSE^ (azure) and PrP^C-BSE^ (green) from brain homogenates. 10^−4^ brain tissue dilutions were used to seed quadruplicate RT-QuIC reactions. Normal control brain homogenates (black) showed no response. The number of samples is in parentheses. (**F**) Representative sensitivity of detection for PrP^C-BSE^ (shades of green) and PrP^L-BSE^ (shades of azure) in brain homogenates (BH) using rHaPrP^Sen^ 90–231 as a substrate. Dilutions are indicated next to the curve. Similar results were obtained from two additional C-BSE-infected and two additional L-BSE-infected brain specimens (presented in Supplementary Fig. S3). Each ThT reading is indicated as the percentage of the maximum value achievable by the plate readers as a function of reaction time.

Chimeric hamster-sheep (Ha-S) rPrP^Sen^ was first used as substrate to evaluate RT-QuIC for the detection of different goat prion strains seeding activity in brain samples. A rapid increase in ThT fluorescence, indicative of newly formed seeded rPrP amyloid fibrils, was observed in each quadruplicate reaction seeded with 10^−4^ TSE-infected brain tissue dilutions within 24 h (Fig. 2A). As previously observed for brain homogenate samples from cattle^33^, the average fluorescence increase was stronger and faster for the L-BSE-infected samples, showing positive signals over the control samples as early as 5 h, compared to the 10–15 h it took for the BSE and scrapie-infected samples. Although the three goat prion strains exhibited distinct RT-QuIC seeded polymerization kinetics, our results show that Ha-S rPrP^Sen^ supports detection of goat C-BSE-, L-BSE- and scrapie-associated seeding activity.

Next, RT-QuIC end-point dilution analysis of three representative clinical TSE-infected goat brain homogenates was performed to evaluate the detection sensitivity for each goat prion strain previously tested. We saw positive reactions with all prion strains using infected brain tissue dilutions down to 10^−8^ (Fig. 2B,D), although reactions seeded with more diluted scrapie samples (10^−8^) were positive only by 40 h incubation (Fig. 2D). This sensitivity was similar to that reported previously for rodent and human prion samples^14^. Spontaneous (unseeded) amyloid formation (rPrP^spon^) was observed in only one of the quadruplicate reactions seeded with normal brain tissue dilutions at 70 h (data not shown). Therefore, we chose 60 h as the reaction cut-off.

In order to explore the prion strain properties of goat C-BSE and L-BSE we tested their relative abilities to initiate polymerization of hamster 90–231 rPrP^Sen^ substrate. RT-QuIC reactions were seeded with 10^−4^ to 10^−8^ brain tissue dilutions and incubated for 60 h using the same experimental conditions described for Ha-S rPrP^Sen^. Both goat BSE prion strains induced conformational conversion of Ha rPrP^Sen^ 90–231 giving the fastest response reactions with the L-BSE seed (Fig. 2E). Conversely to cattle C-BSE^33^, goat C-BSE was able to seed conversion of hamster 90–231 rPrP^Sen^ substrate under these conditions although the sensitivity was limited to less diluted brain tissue homogenates (Fig. 2F).

### High diagnostic value of IQ-CSF across the broad spectrum of small ruminant prions

**Figure 3:**
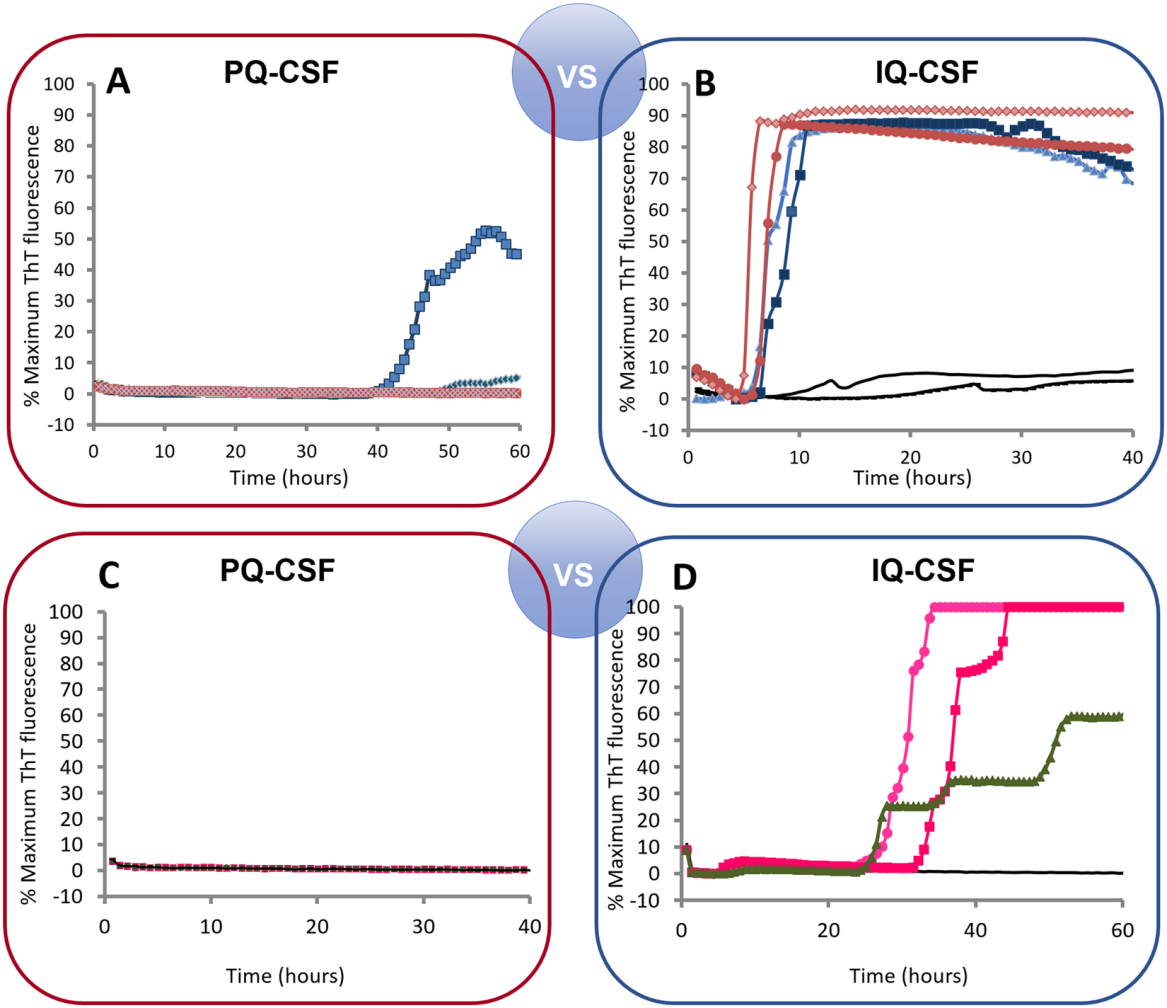
Comparison of first (PQ-CSF) and second generation (IQ-CSF) of RT-QuIC assays using rHaPrP^Sen^ 90–231. (**A,B**) Two L-BSE-infected (G3 and G4, blue) and uninfected (black) goat CSF were tested at 55 °C by using truncated rHaPrP^Sen^ 90–231 with (right) or without (left) the addition of 0.002% SDS. Two human CSF (red) from patients affected with sCJD were used as positive control. Distinct symbols represent separate samples. (**C,D**) Next, rHaSPrP^Sen^ 90–231 substrate was used to detect both PrP^C-BSE^ (green) and PrP^scrapie^ (magenta) from CSF of 3 symptomatic goats (G2, Sc1 and Sc2) using PQ-CSF (**C**) or IQ-CSF (**D**) of RT-QuIC assays.

PQ-CSF and IQ-CSF performances were compared in order to develop experimental conditions for *ante-mortem* detection of prion-infected small ruminants. As previously reported for RT-QuIC-based sCJD diagnosis using CSF samples and N-terminally truncated hamster rPrP^Sen^ (90–231), we found initially that when pure goat CSF was analyzed, positive reactions occurred only after 40 h. However, pure CSF specimens lack SDS that was present in the previously studied brain homogenates, so we then applied RT-QuIC using the combination of the rHaPrP^Sen^ 90–231 substrate, 55 °C, and 0.002% SDS (referred to here as improved QuIC-CSF [IQ-CSF] conditions) to an initial panel of CSF samples collected from two symptomatic L-type BSE-infected goats and two patients affected with sCJD (compare Fig. 3A,B). Our findings indicated that we can sensitively detect PrP^Sc^ associated seeding activity using IQ-CSF in less than 10 h.

Next, we also tested the effects of IQ-CSF conditions on CSF-seeded reaction mixtures using each goat prion strain and Ha 90–231 rPrP^Sen^ as substrate. Notably, 3 of the 3 samples that did not give positive responses under the PQ-CSF conditions (Fig. 3C), gave strong responses using the IQ-CSF conditions (Fig. 3D), while samples from non-TSE control cases remained negative. These findings provided initial evidence that these new reaction conditions improve the speed and diagnostic sensitivity of the RT-QuIC CSF assay for prion goat diseases while maintaining full specificity.

### Early Detection of PrP^Sc^ in CSF of asymptomatic L-BSE infected goats by IQ-CSF

**Figure 4:**
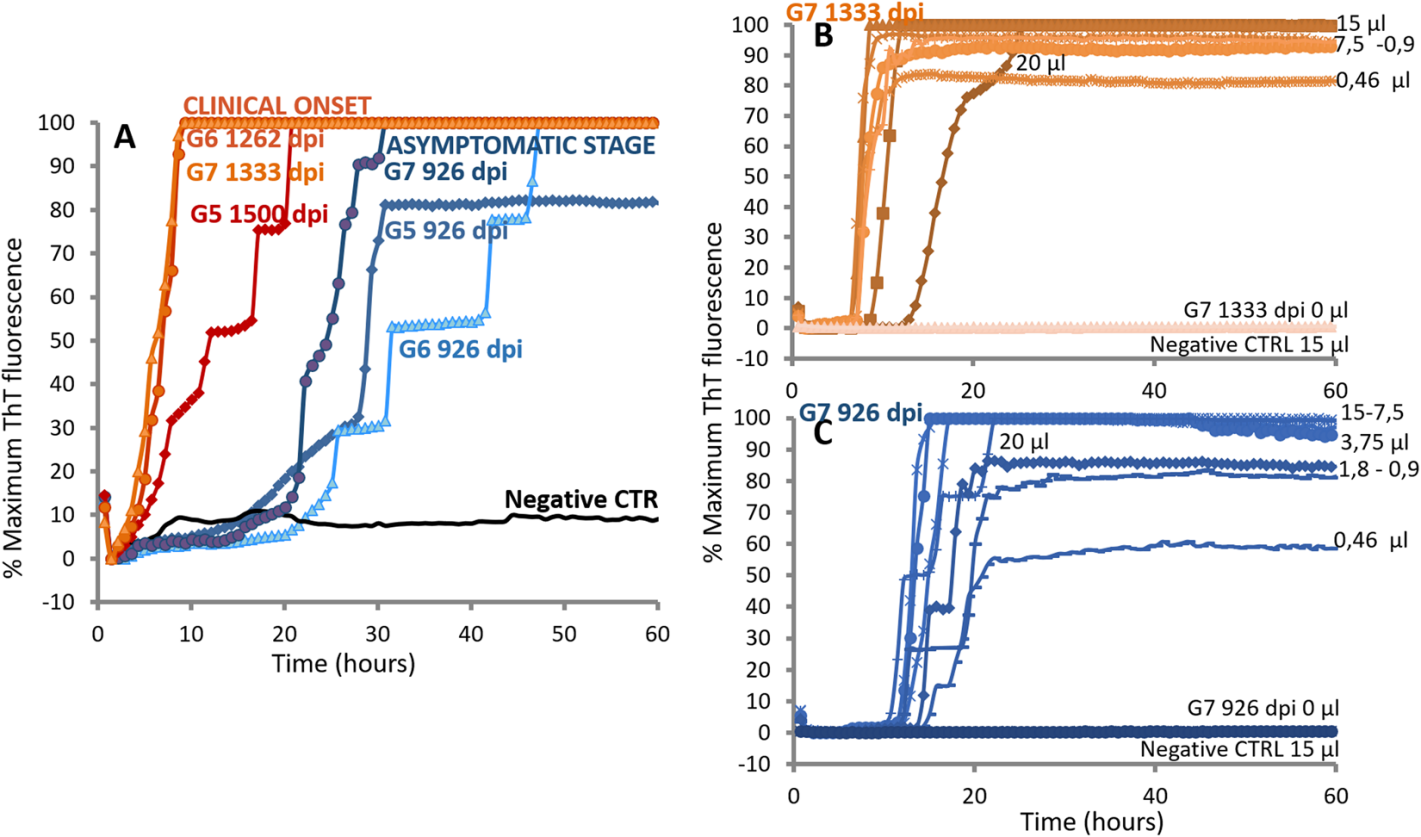
Detection of pathological prion protein in CSF of L-BSE infected goats at the asymptomatic stage using IQ-CSF assay. (**A)** PrP^Sc^ detection in CSF of three L-BSE infected, but asymptomatic goats (shades of blue). CSF collected at 926 days post inoculum (dpi), and at clinical onset (shades of orange) from the same L-BSE-infected goat were tested at 55 °C by using truncated rHaPrP^Sen^ 90–231 with the addition of 0.002% SDS. Distinct symbols represent separate L-BSE samples. (**B)** analytical sensitivity of one goat L-BSE CSF collected at the clinical onset using IQ-CSF conditions. Reaction mixtures seeded with serial decreasing volume of L-BSE-infected CSF samples (20- to 0.46 µl equivalents of pure CSF) were tested. Distinct symbols represent separate volume of pure CSF seeded. (**C)** analytical sensitivity of goat L-BSE-infected CSF collected 407 days before clinical symptoms appeared using IQ-CSF conditions. Reaction mixtures seeded with serial decreasing volume of L-BSE-infected CSF samples (20- to 0.46 µl equivalents of pure CSF) were tested. Distinct symbols represent separate volume of pure CSF seeded.

To investigate the potential of RT-QuIC to detect seeding activity at early time points after inoculation we applied IQ-CSF RT-QuIC to goat CSF collected from 3 L-BSE-infected goats during the pre-symptomatic stage (Table 2). Highly efficient detection of PrP^L-BSE^ associated seeding activity was revealed with an average time of 439 days before clinical symptoms appeared (Fig. 4A).

**Table 2:** Preclinical timepoints at which CSF samples were collected, Survival time (SV), incubation period (IP) and IQ-CSF analysis in L-BSE infected goats.

ID	Preclinical timepoints at which CSF samples were collected (dpi)	SV (dpi)	IP (dpi)	Earlier Preclinical stage in which PrP^Sc^ was detected by IQ-CSF (dbo)
G5	926, 1080, 1200	1597	1500	**574**
G6	926, 1080	1354	1262	**336**
G7	926, 1200	1506	1333	**407**
CTR 2	926, 1080, 1200	—	—	—

dpi = days post inoculum; dbo = days before onset.

Next, we compared the analytical sensitivities of RT-QuIC using IQ-CSF conditions on CSF samples collected at clinical onset and during pre-symptomatic stage that gave positive reactions in the above-described analyses. Four replicate reactions seeded with serial decreasing volume of 2 L-BSE CSF samples (20- to 0.46 µl equivalents of pure CSF) were tested. These measurements revealed equivalent analytical sensitivity between CSF samples collected at clinical onset and more than one year before clinical symptoms appeared. In each case positive reactions occurred until to 0.46 µl of pure CSF seeded (Fig. 4B,C).

**Figure 5:**
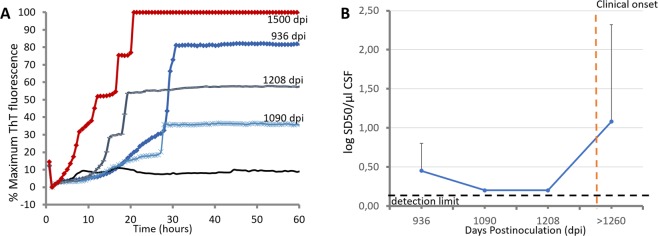
Time course of prion seeding activity detected in CSF of L-BSE-infected goats after intracerebral inoculations. (**A**) Prion seeding activities detected in CSF samples collected at 926 days post inoculum (dpi), 1080 dpi, 1200 dpi, and at clinical onset from L-BSE-infected goat G5. (**B**) Log SD50 analysis of CSF collected from-L-BSE infected goats (G5, G6 and G7) at different time points of asymptomatic stage as indicated in Table 2. Spearman-Karber analysis of the data was used to calculate the dilution of pure CSF required to give 50% positive replicate wells under IQ-CSF conditions (the 50% seeding dose [SD50]). Data are displayed as the mean + the standard deviation.

Furthermore, IQ-CSF conditions were used to evaluate time course of prion accumulation in the CSF of L-BSE-infected goats. Prion seeding activities detected in CSF samples collected 926 days post inoculum (dpi), 1080 dpi, 1200 dpi, and at clinical onset are showed in Fig. 5A. To directly compare the amount of PrP^Sc^ in each time point before clinical onset, we tested endpoint serial dilutions of these CSF samples collected from 2 L-BSE-infected goats. Finally, using Spearman-Karber analysis of the data, we estimated the dilution of pure CSF required to give 50% positive replicate wells under IQ-CSF conditions (the 50% seeding dose [SD50]). Initial Log SD_50_ per µl of CSF was measured at 926 dpi with an average value of 0.45. Soon thereafter, seeding activity began a nearly log-linear decrease down to a minimum of 0.2 LogSD50/µl CSF (Fig. 5B). This level of seeding activity was maintained during the remainder of the asymptomatic stage and appeared clearly increased at clinical onset.

## Discussion

Identification of scrapie or BSE status in goats can be complicated due to the existence of several polymorphisms in the *PRNP* coding sequence which can delay the incubation period and PrP^Sc^ accumulation^34–36^. A recent study by Madsen-Bouterse *et al*.^37^ revealed that the polymorphisms in caprine *PRNP* can also affect the sensitivity of PrP^Sc^ detection in brain samples by anti-prion mAb-based immunoassays such as IHC and Western blot analysis. It therefore becomes important to have a rapid, sensitive, *PRNP* polymorphism-independent as well as antibody-independent detection test to diagnose goat TSE. A previous study already demonstrated sensitive and specific detection of classical scrapie prions in goat brains with different genotypes by RT-QuIC^38^ but to date there is no information on experimental conditions to detect BSE prion seeding activity in small ruminants. We showed that RT-QuIC can be applied successfully to a variety of brain samples from experimental and natural TSE-infected goats including scrapie and BSE. Our findings indicate that by using the Ha-S rPrP^Sen^ substrate, RT-QuIC assays can sensitively detect both C-BSE- and L-BSE-associated seeding activity in less than 24 h. Furthermore, conversely to cattle C-BSE^33^, goat C-BSE prion strain was able to seed conversion of hamster 90–231 rPrP^Sen^ substrate. These data confirm that RT-QuIC can also be used in prion strain typing, as different strains show slight differences in RT-QuIC response^26,33,39–42^.

In fact, one of the main problems related to the management of animal TSEs is the lack of rapid and sensitive tests for *ante-mortem* diagnosis. In small ruminants TSE is suspected diagnostically on the basis of clinical examination of symptomatic individuals and is confirmed *post-mortem* by neuropathological analysis and immunochemical detection of PrP^Sc^ in the central nervous system^12^. One important limitation to this approach is the sensitivity of the detection system, because amounts of PrP^Sc^ high enough to be revealed by conventional methods are only present in the brain at late stages of the disease. However, animal transmission studies show that infectivity is present at a relatively early stage of the incubation period and gradually increases as the disease progresses^43,44^. Our data indicate that RT-QuIC can amplify otherwise undetectable quantities of PrP^Sc^ from CSF of symptomatic and asymptomatic goats. Here, we demonstrate, for the first time, that using recombinant hamster 90–231 PrP^Sen^ as substrate in combination with IQ-CSF, the diagnostic sensitivity of RT-QuIC was clearly improved in CSF samples of both natural scrapie and experimental BSE infected goats, allowing rapid and specific prion seeded polymerization in less than 40 hours. One great advantage of this improved assay is that using hamster 90–231 rPrP^Sen^ substrate which can be universally applied to various animals with different PrP sequences^45^ could avoid the potential problem of polymorphism in sheep or goat. Furthermore, the longitudinal study on experimental L-BSE goats showed that as early as two years and half after infection, i.e., at two fifths of the incubation time, it is possible to distinguish infected from non-infected animals by IQ-CSF analysis. In particular, highly efficient detection of PrP^L-BSE^ associated seeding activity was revealed respectively 574, 336 and 407 days prior to onset of clinical signs. Also, the titration of seeding activity in Fig. 4C showed very high seeding activity in CSF collected at 926 dpi, which suggests that this assay could be able to detect positive samples much earlier than 926 dpi. Moreover, Spearman-Karber analysis indicated that detectable but lower amount of PrP^Sc^ was maintained during the remainder of the asymptomatic stage. Similar time course in brain PrP deposition was observed from pre-clinical and clinical BSE cases reported by Bannach *et al*.^46^. Collectively, these data provide the first indications of the kinetics of prion accumulation in the CSF of prion-infected goats. The observation that PrP seeding activity can be detected in the CSF during early preclinical stages of the disease is consistent with a previous report showing positive RT-QuIC signal in the CSF of intracerebrally scrapie-infected hamsters before the clinical onset^47^. Clearly, the accumulation time course was highly dependent upon the route of prion inoculation. Indeed, the same previous study already demonstrated that after intralingual scrapie inoculation, seeding activity appeared in hamsters CSF only with the onset of clinical signs, well after higher-level accumulation of seeding activity in CSF of intracerebral challenged animals^47^. However, data from recent study on sheep CSF of preclinical and clinical naturally occurring scrapie confirmed that alterations in PrP levels and conformation are primary events in the pathology of prion diseases preceding neuronal damage.

Scrapie is still widespread, and the only available eradication measure is the genotype-based eradication program. However, this plan only shows effects in years and requires a high level of compliance by farmers, which is not always the case. Although, furthers studies are necessary to assess the ability of RT-QuIC to detect prion seeding activity in biological samples of more diagnostic interest as blood and excreta, the *ante-mortem* CSF test here described is one of few *ante-mortem* screening methods now available to detect asymptomatic TSE-infected goats^48^. It could represent a more rapid and sensitive approach to identify TSE-infected flocks, assaying symptomatics and all genetically susceptible animals. Infected animals could be eliminated at an early stage, with a rapid decrease of the potential environmental infection load. Such a surveillance system will also allow decreases the genetic selection pressure, maintaining some genetic variability in the populations, as desired by farmers.

Overall, our data suggest that CSF prion seeding activity may be related to early prion pathological events and may allow the design of a diagnostic test for preclinical and clinical animals, minimizing the risk of potential exposure to others in the herd.

## Materials and Methods

### Study Populations and Sample Collection

The analyses with RT-QuIC were carried out on brainstem and CSF samples collected from goats derived from two natural outbreaks of classical scrapie and from caprines intracranially inoculated with 1 ml of a 10% (wt./vol.) brain homogenate from a cow diagnosed with classical (C-BSE) or atypical BSE (L-BSE) as previously described^11^. The physical and neurological status of the animals was monitored monthly by a board certified veterinary neurologist. For this purpose, a clinical examination protocol, previously used for sheep^49^ was applied. CSF was collected from the lumbosacral site of each animal, while it was standing, as described by Mayhew *et al*.^50^. The animal was sedated with 0.05 mg/Kg xylazine (Rompun ®, Bayer Health care) IV. The collection site (7 × 7 cm) was clipped, surgically prepared and locally anaesthetized with 2.0 ml of procaine hydrochloride (Aticain®, Ati S.r.l.). Disposable 21 G 0.80 mm × 50.00 mm hypodermal needles (Terumo ®) were used to collect 5 mL of CSF by gentle syringe aspiration into a sterile tube (Uridraw, Terumo ®). In the presence of symptomatology related to the TSE, the animals were euthanized with i.v. enbutramide/mebezonium iodide/tetracaine hydrochloride (Tanax®, Intervet Inc. Merck). After euthanasia, CSF, the whole brain, the entire spinal cord and peripheral nervous tissues were sampled. Each sample was frozen at −80 °C.

### PrP^Sc^ extraction and confirmatory Western blot (WB) analysis

PrP^Sc^ extraction and the following WB analyses were carried out on the brainstem samples collected at necropsy from the experimentally challenged goats. Brain tissue from healthy cattle, C-BSE and L-BSE samples, used for the *inoculum*, and goats previously confirmed positive or negative for classical scrapie were also analyzed as controls. The analyses were performed as previously reported^11^. PrP^Sc^ was detected using SAF84 (mouse; Cayman Chemical Co.; Cat. No. 189775) monoclonal antibody and an anti-mouse antiserum conjugated with alkaline phosphatase. Reaction was revealed by a chemiluminescent substrate and visualized on Hyperfilm ECL (Amersham GE-Healthcare, Life Sciences) or by a gel documentation (UVI Prochemi, Uvitec). Image Lab^TM^ analysis software (Bio-Rad) was used to analyse blot images.

### Recombinant prion protein purification

Syrian golden hamster residues 90 to 231 (hamster 90–231 or Ha 90–231) and chimeric hamster-sheep (Ha-S; Syrian hamster residues 23 to 137 followed by sheep residues 141 to 234 of the R_154_Q_171_ polymorph [accession no. AY907689]) prion protein genes were ligated into the pET41 vector (EMD Biosciences). *Escherichia coli* carrying this vector was grown in Luria broth (LB) medium in the presence of kanamycin and chloramphenicol. rPrP^Sen^ expression was induced using Overnight Express Autoinduction system 1 (Novagen) and Bug Buster master mix (Novagen) to isolate inclusion bodies. Following solubilisation of the inclusion bodies in 8M guanidinium-HCl, the denatured protein was purified under 6M guanidinium-HCl denaturing conditions using nickel nitrilotriacetic acid (Ni-NTA) superflow resin (Qiagen) with an AKTA 25 L protein liquid chromatography instrument (GE-Healthcare, Life Sciences). The rPrP^Sen^ was subjected to on-column refolding using a linear gradient into phosphate buffer and then eluted using an imidazole gradient as previously described^14^. The purified protein was extensively dialyzed into 10 mM sodium phosphate buffer (pH 5.8). Then, following filtration (0.22-μm syringe filter; Sartorius), a concentration measurement by absorbance at 280 nm was performed and the rPrP^Sen^ was stored at −80 °C.

### Brain homogenates preparation and RT-QuIC protocol

Normal (*n* = 2), scrapie-infected (n = 2), C-BSE-infected (*n* = 2), and L-BSE-infected (*n* = 2) goat brain homogenates (BHs) (10%, wt/vol) were prepared as previously described^33^ and stored at −80 °C. For RT-QuIC analysis, BHs were serially diluted in 0.1% SDS (Sigma)–N2 (Gibco)–PBS as previously reported^14^. The RT-QuIC reaction mix was composed of 10 mM phosphate buffer (pH 7.4), 300 mM NaCl, 10 μM ThT, 1 mM EDTA, and 0.1 mg/ml of rPrP^Sen^. Aliquots of this mix (98 μl) were loaded into each well of a black 96-well plate with a clear bottom (Nunc) and seeded with 2 μl of 10^−4^ to 10^−9^ brain homogenate dilutions. Normal goat BH dilutions were used as negative controls (as shown in Fig. 2), and 10^−4^ brain homogenate dilutions from bovine or sheep with clinical BSE or scrapie, respectively, were initially included as positive controls when goat brain samples were tested for the first time. The plate was then sealed with a plate-sealer film (Nalgene Nunc International) and incubated for 70 h at 42 °C in a BMG Labtech FLUOstar plate reader with cycles of 1 min of shaking (700 rpm double orbital) and 1 min of rest throughout the incubation. ThT fluorescence measurements (excitation, 450 ± 10 nm; emission, 480 ± 10 nm; bottom read) were recorded every 45 min. RT-QuIC reactions were deemed acceptable when the negative controls remained negative for at least 50 h.

### CSF preparation, PQ-CSF and IQ-CSF protocols

Native CSF samples were centrifuged at 1000 × g for 10 min to remove cell debris, using 5417 R Eppendorf centrifuge (or an equivalent). Supernatant was transferred to new 1.5-ml tubes and stored at −20 °C. IQ-CSF assays were performed as reported previously for sCJD patients^25^. Briefly, both PQ-CSF and IQ-CSF reactions were run with rPrP^Sen^ Ha 90–231 with or without the addition of 0.002% SDS to the reaction mix.

### Data analysis

RT-QuIC fluorescence readings were analyzed as previously described^33^. Briefly, to compensate for differences between the fluorescence plate readers, data sets were normalized to a percentage of the maximal fluorescence response of the instrument, and the obtained values were plotted against the reaction times. Samples were judged to be RT-QuIC positive using criteria similar to those previously described for RT-QuIC analyses of brain specimens^14,33^. A 60 h time point was chosen based on multiple (*n* = 20) repeat runs in which no spontaneous conversions of the substrate in negative-control seeded reactions were observed up to the experimentally designated time point. Data are displayed as the average of four technical replicates except where indicated. Where multiple biological replicates are displayed, they are represented as the mean ± the standard deviation.

### Ethics Statement

All procedures involving animals and their care were conducted in conformity with national and international laws and policies (EEC Council Directive 86/609, 63/2010; Italian Legislative Decree 116/92 and 26/2014). The study was approved by the Italian Ministry of Health with authorization number 694/2015-PR of 17th of July 2015.

## Supplementary information

Supplementary Figures
