# Supplementary material for: Early and Pre-Clinical Detection of Prion Seeding Activity in Cerebrospinal Fluid of Goats using Real-Time Quaking-Induced Conversion Assay
Source: Sci Rep. 2019 Apr 16;9:6173. doi: 10.1038/s41598-019-42449-7 (PMC6467873; doi:10.1038/s41598-019-42449-7)
Supplement: Supplementary file 1 — Supplementary Figures [file 41598_2019_42449_MOESM1_ESM.docx]

**Supplementary materials**

Early and Pre-Clinical Detection of Prion Seeding Activity in Cerebrospinal Fluid of Goats using Real-Time Quaking-Induced Conversion Assay

*Alessandra Favole^1^, Maria Mazza^1^, Elena Vallino Costassa^1^, Antonio D’Angelo^2^, Guerino Lombardi^3^, Paola Marconi^4^, Paola Crociara^1^, Elena Berrone^1^, Marina Gallo^1^, Claudia Palmitessa^1^, Christina D. Orrù^5^, Byron Caughey^5^, Pier L. Acutis^1^, Maria Caramelli^1^, Cristina Casalone^1^ and Cristiano Corona^1,*^*

^1^ National Reference Laboratory of TSEs (CEA), Istituto Zooprofilattico Sperimentale del Piemonte, Liguria e Valle d'Aosta, Turin, Italy.

^2^ Dipartimento di Scienze Veterinarie, Sezione Clinica Medica, University of Turin, Grugliasco (Turin), Italy.

^3^ Istituto Zooprofilattico Sperimentale della Lombardia e dell'Emilia Romagna, Brescia, Italy.

^4^ Istituto Zooprofilattico Sperimentale Lazio e Toscana, Firenze, Italy

^5^ Rocky Mountain Laboratories, National Institute for Allergy and Infectious Diseases, National Institutes of Health, Hamilton, Montana, USA

***** Corresponding author

E-mail: [cristiano.corona@izsto.it](mailto:cristiano.corona@izsto.it)

**
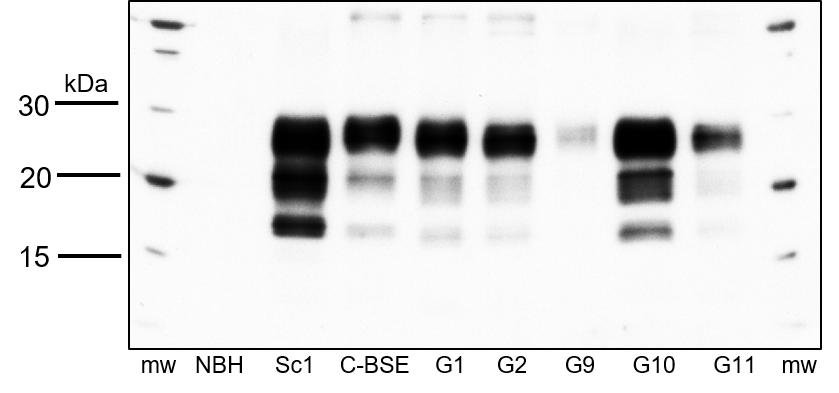
**

**Supplementary Figure S1. Original western blot for the cropped image used in Figure 1A.** Region of western blot shown in the figure 1A was highlighted in red boxe. mw: molecular weights; NBH: normal brain homogenate; Sc1: classical scrapie goat; G1, G2, G9-11: goats inoculated with C-BSE. All samples were treated with Proteinase K. Membrane was probed with mAb SAF 84.

**
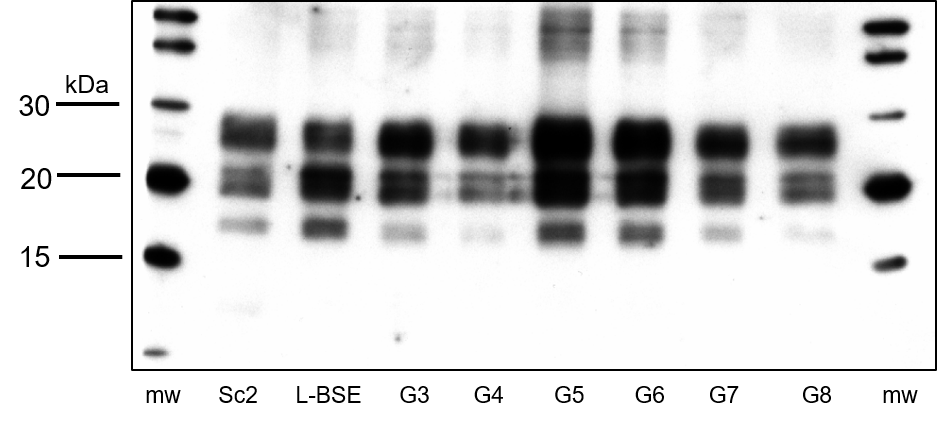
**

**Supplementary Figure S2. Original western blot for the cropped image used in Figure 1B.** Region of western blot shown in the figure 1B was highlighted in red boxe. mw: molecular weights; Sc2: classical scrapie goat; G3-G8: goats inoculated with L-BSE. All samples were treated with Proteinase K. Membrane was probed with mAb SAF 84.

**
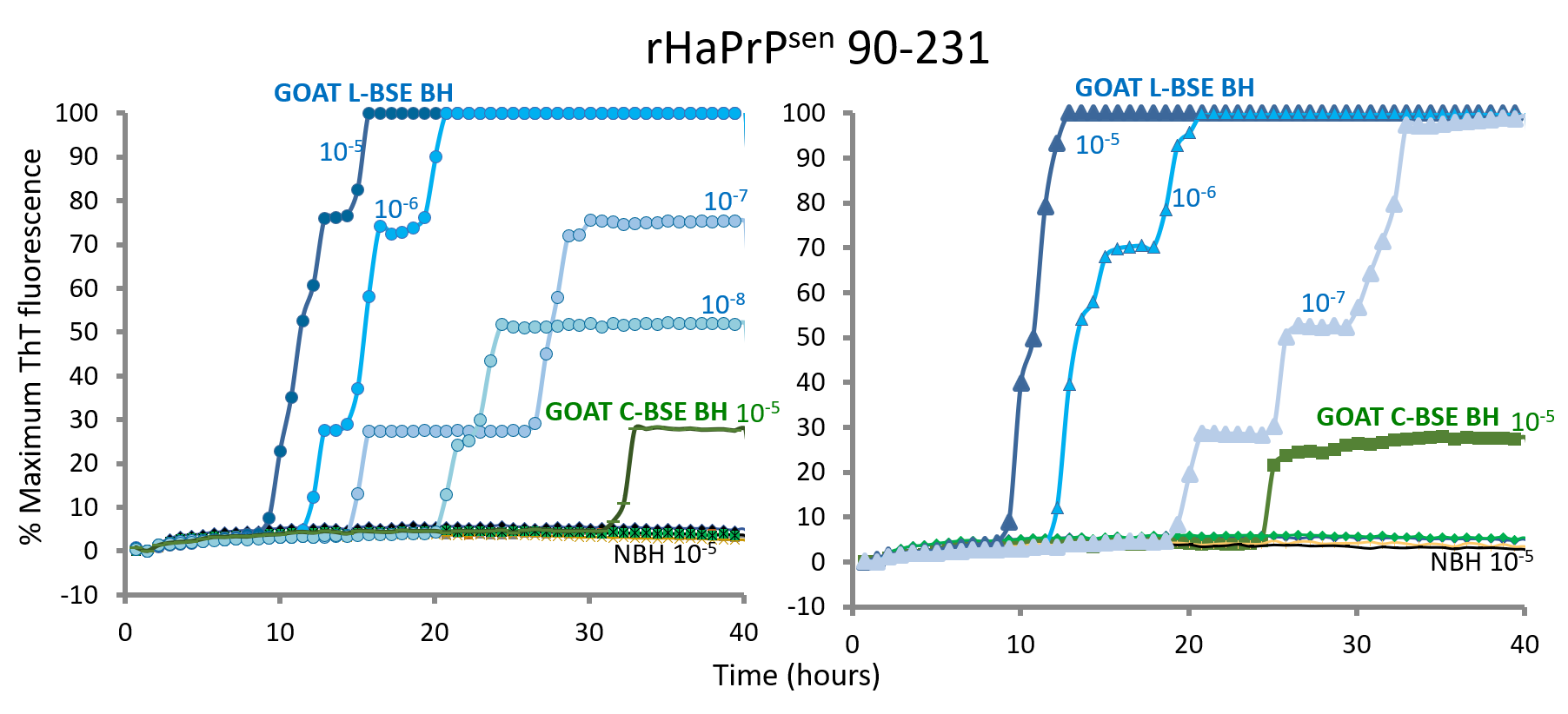
**

**Supplementary Figure S3.** Serial dilutions (10^−5^ to 10^−8^) of C-BSE-infected (shades of green) or L-BSE-infected (shades of blue) brain tissue or a 10^−5^ dilution of uninfected brain tissue (black) were used to seed quadruplicate RT-QuIC reactions with Ha 90-231 rPrP^Sen^ as the substrate. The data show the average ThT fluorescence of 4 replicate wells. Distinct symbols represent separate sample of brain homogenate seeded. Dilutions are indicated next to the curve.
